# Doppler flow morphology characteristics of epiaortic arteries in aortic valve pathologies: a retrospective study on a cohort of patients with ischemic stroke

**DOI:** 10.1186/s13089-023-00327-4

**Published:** 2023-06-07

**Authors:** Stefanie Meyer, Lara Wilde, Frieder Wolf, Jan Liman, Mathias Bähr, Ilko L. Maier

**Affiliations:** 1 Department of Neurology, University Medicine Göttingen, Robert-Koch-Str. 40, 37075 Göttingen, Germany; 2 Department of Cardiology, University Medicine Göttingen, Göttingen, Germany; 3 Department of Neurology, Paracelsus Medical University Nuremberg Hospital, Nuremberg, Germany

**Keywords:** Doppler sonography, Stroke, Aortic valve regurgitation, Aortic valve stenosis

## Abstract

**Background and aims:**

Neurovascular ultrasound (nvUS) of the epiaortic arteries is an integral part of the etiologic workup in patients with ischemic stroke. Aortic valve disease shares similar vascular risk profiles and therefore not only presents a common comorbidity, but also an etiologic entity. The aim of this study is to investigate the predictive value of specific Doppler curve flow characteristics in epiaortic arteries and the presence of aortic valve disease.

**Methods:**

Retrospective, single-center analysis of ischemic stroke patients, both receiving full nvUS of the extracranial common- (CCA), internal- (ICA) and external carotid artery (ECA) and echocardiography (TTE/TEE) during their inpatient stay. A rater blinded for the TTE/TEE results investigated Doppler flow curves for the following characteristics: ‘pulsus tardus et parvus’ for aortic valve stenosis (AS) and ‘bisferious pulse’, ‘diastolic reversal’, ‘zero diastole’ and ‘no dicrotic notch’ for aortic valve regurgitation (AR). Predictive value of these Doppler flow characteristics was investigated using multivariate logistic regression models.

**Results:**

Of 1320 patients with complete examination of Doppler flow curves and TTE/TEE, 75 (5.7%) showed an AS and 482 (36.5%) showed an AR. Sixty-one (4.6%) patients at least showed a moderate-to-severe AS and 100 (7.6%) at least showed a moderate-to-severe AR. After adjustment for age, coronary artery disease, arterial hypertension, diabetes mellitus, smoking, peripheral arterial disease, renal failure and atrial fibrillation, the following flow pattern predicted aortic valve disease: ‘pulsus tardus et parvus’ in the CCA and ICA was highly predictive for a moderate-to-severe AS (OR 1158.5, 95% CI 364.2–3684.8, *p* < 0.001). ‘No dicrotic notch’ (OR 102.1, 95% CI 12.4–839.4, *p* < 0.001), a ‘bisferious pulse’ (OR 10.8, 95% CI 3.2–33.9, *p* < 0.001) and a ‘diastolic reversal’ (OR 15.4, 95% CI 3.2–74.6, *p* < 0.001) in the CCA and ICA predicted a moderate-to-severe AR. The inclusion of Doppler flow characteristics of the ECA did not increase predictive value.

**Conclusions:**

Well defined, qualitative Doppler flow characteristics detectable in the CCA and ICA are highly predictive for aortic valve disease. The consideration of these flow characteristics can be useful to streamline diagnostic and therapeutic measures, especially in the outpatient setting.

**Supplementary Information:**

The online version contains supplementary material available at 10.1186/s13089-023-00327-4.

## Introduction

Extracranial neurovascular ultrasound (nvUS) of the carotid arteries is one of the most frequently performed diagnostic procedures in neurological in- and outpatient departments and is a state-of-the-art investigation in all patients suffering from ischemic stroke [1]. Aortic valve disease (e.g., aortic valve regurgitation (AR) and aortic valve stenosis (AS)) presents a relevant and frequent comorbidity in stroke patients due to shared risk factor profiles [2]. If detected, effective surgical and minimally invasive treatments are available to improve cardiac valve function [3, 4].

Both, AR and AS, can alter the flow characteristics detectable by neurovascular ultrasound (nvUS) in the brain supplying arteries [5–8]. Under physiological conditions, the common (CCA), internal (ICA), and external (ECA) carotid arteries show characteristic waveforms. The ECA displays high pulsatility due to increased peripheral resistance, whereas ICA shows a less pulsatile flow profile due to a higher constant diastolic blood flow [9–11]. In the CCA, a combination of both flow spectrum characteristics can be found.

In the past, small case series described various Doppler flow curve characteristics associated with aortic valve pathology in the brain supplying vessels [6]. AR has been shown to be associated with different flow profile changes, for example ‘bisferious pulse’, which is characterized by the display of two systolic peaks of similar height (or increased height of the second peak), with a significant flow decrease in between [12]. Appearance of ‘bisferious pulse’ increased with severity of AR [7]. The ‘diastolic flow reversal’ profile describes a flow alteration with negative retrograde flow of equal to or over 50% of diastole duration, caused by the reverse blood flow from the aorta into the left ventricle during diastole [13]. These flow characteristics have been reported to normalize after surgical treatment of AR [6]. ‘Zero level diastolic flow’ (‘zero flow’ / ‘zero diastole’) describes a lack of blood flow during a minimum of 50% of diastolic duration. Another waveform associated with AR is ‘no dicrotic notch’, which is characterized by lack of a dicrotic wave at the end of systole and probably caused by insufficient closure of the aortic valve or alterations in reflected pressure waves associated with AR [14].

In AS, Doppler waveforms show a delayed systolic upstroke and a rounded waveform, described as ‘pulsus tardus et parvus’, which, in a study with 24 patients, has been found in all patients with critical AS (9).

In daily clinical practice, cerebro- and cardiovascular disease are closely linked. This includes relevant valvular pathologies like AS and AR, which are frequently diagnosed in stroke patients [15]. The main aim of this study is the systematic analysis of the predictive value of the previously described qualitative Doppler flow characteristics in nvUS, associated with aortic valve pathology, in a cohort of patients with ischemic stroke. The secondary aim is identification of epiaortic artery sections most suitable for this assessment. Overall goals of the study are to improve streamlining of diagnostics and interpretation of nvUS results, as well as identification of high-risk patients for prioritized echocardiography.

## Methods

### Patient population and study design

**Fig. 1:**
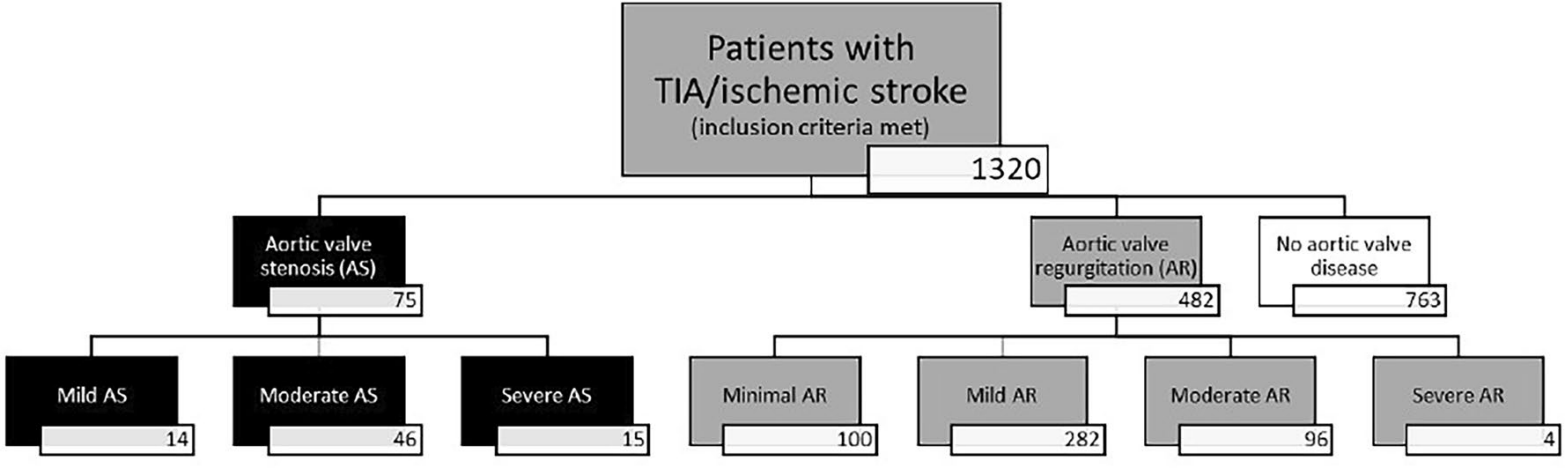
Inclusion flowchart

In this single-center, retrospective observational study, data from patients with an admission diagnosis of transitory ischemic attack (TIA) or ischemic stroke treated in a tertiary university hospital between September of 2015 and March of 2019 were collected. No formal sample size regarding statistical analyses was calculated prior to start of this study, but all eligible patients during the named time-period were screened, and, if fulfilling inclusion criteria, included into the study. Clinical and imaging data regarding cardiovascular risk factors, nvUS and TTE were extracted from digital clinical information systems. When additional transeosophageal echocardiography (TEE) was available, results were included as well. Only patients receiving nvUS and TTE/TEE during in-hospital stay and with sufficient documentation regarding baseline characteristics were included in the study. Patients with stenosis or occlusion of carotid artery were excluded, due to possible flow pattern alterations caused by carotid vessel pathology, independent of valvular disease, with final inclusion of 1320 patients (Fig. 1).

Baseline characteristics, including information on cardiovascular risk factors as well as comorbidities (arterial hypertension, coronary heart disease, previous ischemic stroke, heart failure, hyperlipidemia, renal failure, smoking, obesity, diabetes mellitus and peripheral artery disease (PAD)), age and gender, were obtained from in-hospital documentation (xserv (ixmid software technology GmbH^©^) and the intensive care information system (IntelliSpace Critical Care and Anaesthesia^©^, ICCA (Philips)). Results of stroke-imaging and neurovascular ultrasound, as well as TTE/TEE, were documented.

### Flow pattern analysis

**Fig. 2:**
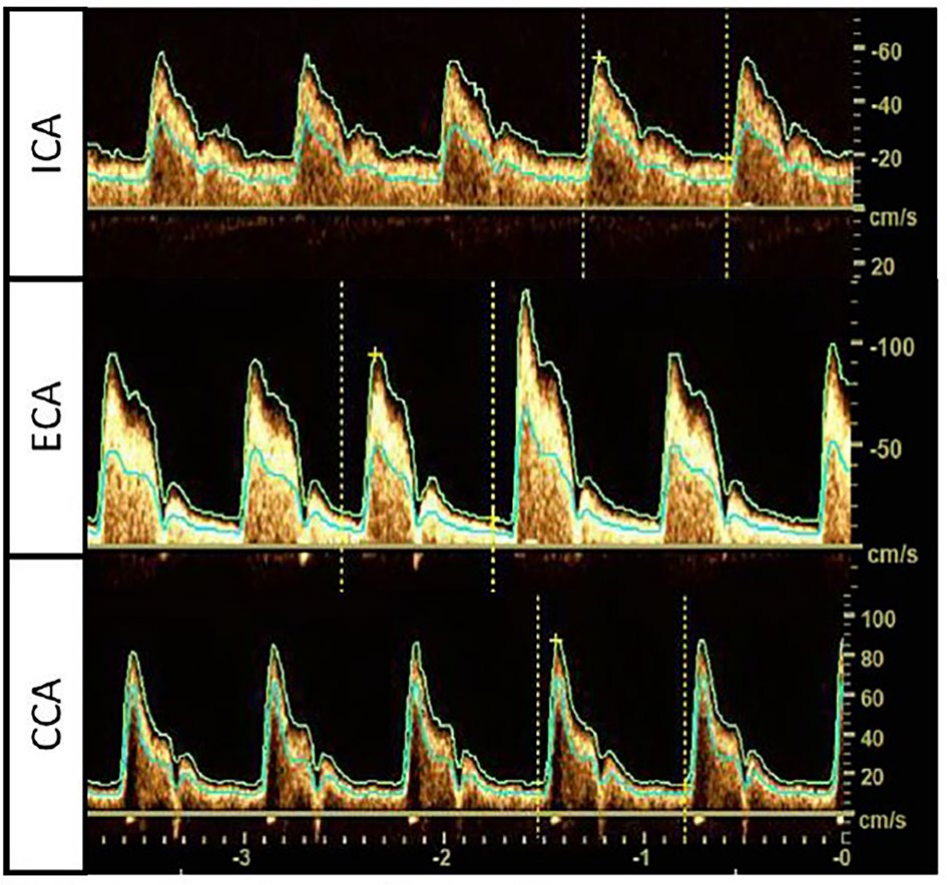
Physiological flow profiles in nvUS. Physiological flow profiles in the various parts of the carotid vessels differ. The normal flow curve in ICA (first panel) is characterized by one systolic peak with less pulsatility due to a higher constant diastolic flow compared to ECA (second panel) and CCA. The last curve shows the more pulsatile flow profile in CCA, presenting with combined characteristics of ICA and ECA flow profiles

Images of routine diagnostic nvUS were evaluated regarding predefined qualitative Doppler flow curve characteristics blinded to the TTE/TEE results (aortic valve status). Representative, physiological flow curves are shown in Fig. 2.

The high pulsatility of the ECA due to an increased peripheral resistance and the less pulsatile flow with higher end-diastolic velocities in the ICA, as well as the mixture of both characteristics in the Doppler flow curves of the CCA, can be noted.

**Fig. 3:**
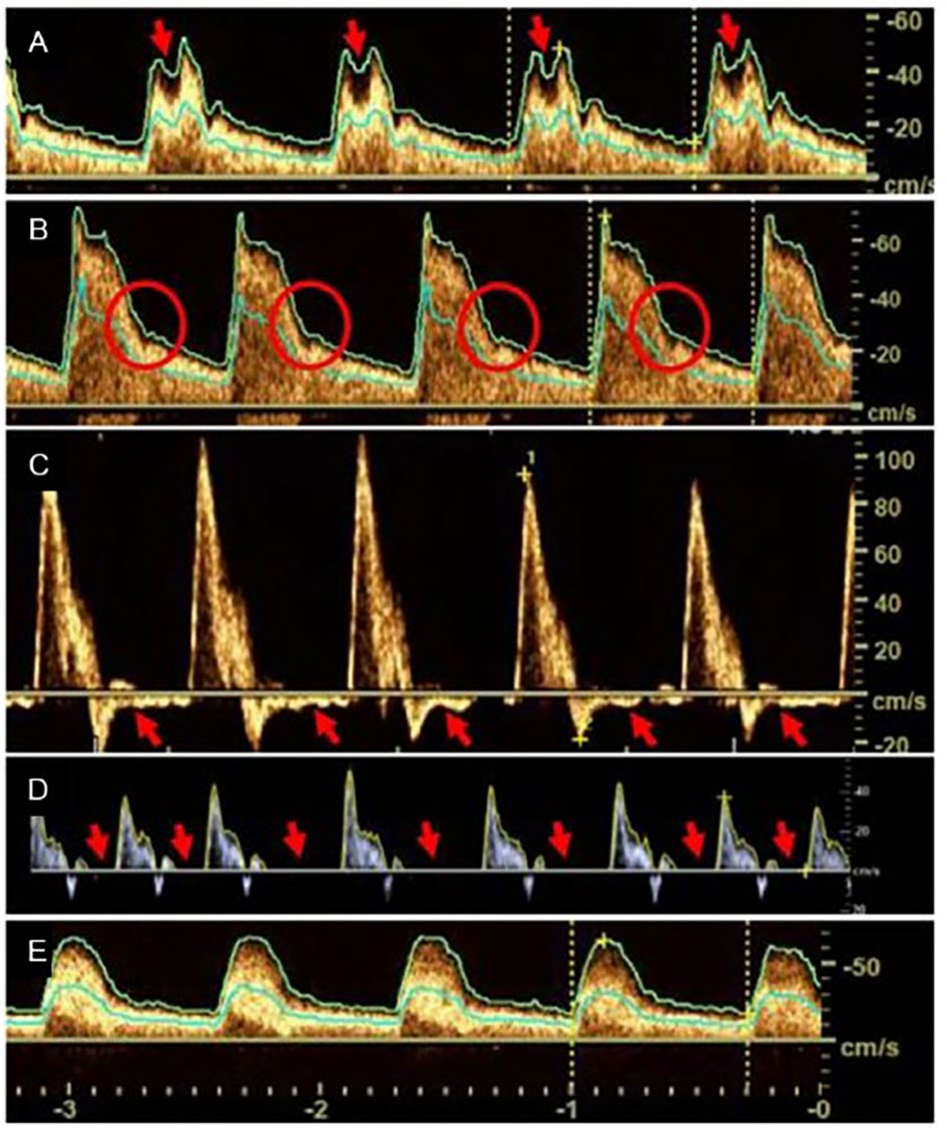
Flow profiles in nvUS associated with aortic valve disease: ‘bisferious pulse’ in the ICA (red arrows, **A**), ‘no dicrotic notch’ in the ICA (red circle, **B**), ‘diastolic reversal’ in the CCA (red arrows, **C**), ‘zero diastole’ (red arrows, **D**) and ‘pulsus tardus et parvus’ in the ICA (**E**)

In contrast, distinct qualitative alterations of the Doppler flow curves have both been identified in patients with AS and AR. Representative flow curve characteristics indicating AR (bisferious pulse, A; no dicrotic notch, B; diastolic reversal, C; and zero diastole, D) and AS (pulsus tardus et parvus, E) are shown in Fig. 3.

Flow alterations indicating AR were classified as ‘no flow changes’, ‘bisferious pulse’, ‘zero flow’, ‘diastolic reversal’ and ‘absence of dicrotic notch’. Flow alterations indicating AS were categorized in ‘no flow changes’ versus appearance of ‘pulsus tardus et parvus’. These flow patterns had to be detectable in one or both sides of the investigated part of the carotid artery. Patients showing CCA, ICA or ECA stenosis in nvUS or CT-angiography of the brain supplying epiaortic arteries were excluded from the study. After completion of the nvUS rating, TTE/TEE examination results were assessed, which were previously categorized by the treating senior cardiologist as follows: ‘no valve pathology’, ‘minimal AR’, ‘mild AR’, ‘moderate AR’, ‘severe AR’ as well as ‘mild AS’, ‘moderate AS’ and ‘severe AS’ according to clinical standard.

### Statistical analysis

Statistical analysis was performed using SPSS 28 (IBM SPSS Statistics, Armonk, NY, USA, 2019). Baseline characteristics were described using frequencies, means and median with a standard deviation and interquartile range, when applicable. Descriptive statistics were performed using Chi-square test. Sensitivity, specificity, positive and negative predictive values were calculated using fourfold tables. Subgroup analysis was performed using exact t-test. Uni- and multivariate logistic regression models were used to calculate prediction probability for single flow variations. For the multivariate, binary logistic regression analysis, the backward selection function with a removal cut-off from the model of *p* < 0.1 was used to identify the factors with the highest predictive value. To evaluate the predictive value of the Doppler flow patterns, odds ratio (OR) and 95% confidence intervals (CIs) were calculated using multivariate logistic regression models. Significance level was determined at *p* < 0.05. Inter-rater reliability was investigated using Cohens kappa (κ).

## Results

### Baseline characteristics

In total, 1320 datasets/patients were included in the study. Cardiovascular risk factors as baseline characteristics in patients with AR were analyzed depending on the severity of the vitium, and patients separated into five groups (no AR, minimal AR, mild AR, moderate AR, and severe AR) accordingly (Table 1). Overall, the most common cardiovascular risk factors in AR were arterial hypertension (73%), previous ischemic stroke (65%), and atrial fibrillation (30.5%). Increased age was significantly correlated with the severity of AR, except for severe AR, which showed a lower patient age. Correlation of cardiovascular risk factors associated with the severity of AR was observed for ischemic stroke, coronary heart disease, heart failure, diabetes mellitus, renal failure, and atrial fibrillation. Hyperlipidemia, obesity, and PAD decreased in frequency with an increasing severity of AR in this patient cohort. The risk factors coronary heart disease, arterial hypertension, diabetes mellitus and atrial fibrillation were more frequent in patients with AR compared to patients without detection of this vitium.

**Table 1:** Baseline characteristics of patients with aortic valve regurgitation (AR)

Clinical parameters	Aortic valve regurgitation (AR) (n = 482)					*P* value
	No AR (*n* = 838)	Minimal AR (*n* = 100)	Mild AR (*n* = 282)	Moderate AR (*n* = 96)	Severe AR (*n* = 4)	
Age (mean ± SD)	67.2 ± 14.8	71.2 ± 12.1	77 ± 10.9	79.5 ± 10.9	71.8 ± 16.9	< 0.001
Gender (*n*, % male)	452 (53.9)	64 (64)	139 (49.3)	41 (42.7)	4 (100)	0.061
Ischemic stroke (*n*, %)	478 (57)	57 (57)	183 (64.9)	70 (72.9)	3 (75)	0.01
Coronary heart disease (*n*, %)	111 (13.2)	14 (14)	53 (18.8)	21 (21.9)	0 (0)	0.05
Heart failure (*n*, %)	62 (7.4)	6 (6)	26 (9.2)	14 (14.6)	2 (50)	0.003
Hypertension (*n*, %)	550 (65.6)	71 (71)	208 (73.8)	69 (71.9)	4 (100)	0.052
Diabetes mellitus type 2 (*n*, %)	167 (19.9)	22 (22)	64 (22.7)	23 (24)	0 (0)	0.609
Hyperlipidemia (*n*, %)	236 (28.2)	30 (30)	73 (25.9)	22 (22.9)	0 (0)	0.495
Nicotine consume (*n*, %)	195 (23.3)	12 (12)	25 (8.9)	11 (11.5)	1 (25)	0.1
Obesity (*n*, %)	95 (11.3)	8 (8)	27 (9.6)	3 (3.1)	0 (0)	0.108
Peripheral artery disease (PAD) (*n*, %)	25 (3)	2 (2)	5 (1.8)	2 (2.1)	0 (0)	0.807
Renal failure (*n*, %)	57 (6.8)	5 (5)	31 (11)	15 (15.6)	0 (0)	0.008
Atrial fibrillation (*n*, %)	136 (16.2)	25 (25)	87 (30.9)	34 (35.4)	1 (25)	0.01

*SD* standard deviation, differences were analyzsed using Chi-square test and ANOVA

% of patients within each group, *n* number of patients, *AR* aortic valve regurgitation

Patients with aortic valve stenosis showed a similar pattern of baseline characteristics compared to patients with AR (Table 2). The most common cardiovascular risk factors were arterial hypertension (85%), previous stroke (69%) and atrial fibrillation (43%). Increase in severity of AS was associated significantly with patients’ age. An increased frequency of heart failure, diabetes and obesity was also associated with severity of AS. Overall, patients with AS showed hyperlipidemia, PAD, renal failure, and arterial hypertension more often than patients without AS, whereas coronary heart disease, diabetes mellitus, smoking, and obesity were more common in patients without AS.

**Table 2:** Baseline characteristics of patients with aortic valve stenosis (AS)

Clinical parameters	Aortic valve stenosis (AS) (*n* = 75)				*P* value
	No AS (*n* = 1245)	Mild AS (*n* = 14)	Moderate AS (*n* = 46)	Severe AS (*n* = 15)	
Age (mean ± SD)	69.8 ± 14.4	80.4 ± 5.9	81.9 ± 8.3	81.9 ± 11.8	< 0.001
Gender (*n*, % male)	659 (53)	6 (42.9)	29 (63)	6 (40)	0.740
Ischemic stroke (*n*, %)	739 (59.4)	10 (71.4)	31 (67.4)	11 (73.3)	0.372
Coronary heart disease (*n*, %)	177 (88.9)	4 (28.6)	16 (34.8)	2 (13.3)	0.001
Heart failure (*n*, %)	99 (90)	1 (7.1)	7 (15.2)	3 (20)	0.122
Hypertension (*n*, %)	838 (67.3)	9 (64.3)	42 (91.3)	13 (86.7)	0.003
Diabetes mellitus type 2 (*n*, %)	252 (91.3)	4 (28.6)	15 (32.6)	5 (33.3)	0.110
Hyperlipidemia (*n*, %)	336 (27)	6 (42.9)	15 (32.6)	5 (33.3)	0.490
Nicotine consume (*n*, %)	238 (19.2)	2 (14.3)	2 (4.3)	2 (13.3)	0.077
Obesity (*n*, %)	128 (10.3)	0 (0)	2 (4.3)	3 (20)	0.178
Peripheral artery disease (PAD) (*n*, %)	29 (2.3)	1 (7.1)	3 (6.5)	1 (6.7)	0.150
Renal failure (*n*, %)	95 (7.6)	3 (21.4)	8 (17.4)	2 (13.3)	0.023
Atrial fibrillation (*n*, %)	251 (20.2)	4 (28.6)	22 (47.8)	6 (40)	0

*SD* standard deviation, differences were analyzed using Chi-square test and ANOVA

% of patients within each group, *n* number of patients, *AS* aortic valve stenosis

### Doppler flow profile characteristics in patients with aortic valve regurgitation

In patients without AR, AR-associated flow profile changes were only detectable in nvUS in singular cases, most often in CCA, less frequent in ICA but never in both vessels (Table 3, full data included in Additional file 1). In minimal AR, only one patient showing nvUS flow profile changes was identified, displaying a ‘bisferious pulse’ in ICA. The frequency of changes in flow profiles increased coherent with the severity of AR. Overall, most changes of flow patterns were identified in CCA. The characteristics ‘bisferious pulse’ and ‘no dicrotic notch’ were found to be more common in CCA than in ICA and their frequency increased associated with the severity of AR (‘bisferious pulse’ in CCA in mild AR 14.2%, moderate AR 21.9%, severe AR 25%, ‘no dicrotic notch’ in CCA 15.6% in mild AR, 31.3% in moderate AR). In severe AR, flow characteristic ‘zero flow’ and ‘diastolic reversal’ appeared additionally in 50% of cases in the CCA, whereas these flow changes were only detected in a small percentage of patients with mild or moderate AR.

**Table 3:** nvUS flow characteristics in patients with AR

Flow characteristic	No AR (*n*, %) *N* = 838	Minimal AR (*n*, %) *N* = 100	Mild AR (*n*, %) *N* = 282	Moderate AR (*n*, %) *N* = 96	Severe AR (*n*, %) *N* = 4	*P* value
Bisferious pulse CCA	5 (0.6)	0 (0)	40 (14.2)	21 (21.9)	1 (25)	< 0.001
Bisferious pulse ICA	2 (0.2)	1 (1)	27 (9.6)	18 (18.8)	1 (25)	< 0.001
Bisferious pulse CCA + ICA	0 (0)	0 (0)	7 (2.5)	5 (5.2)	1 (25)	< 0.001
Zero diastole CCA	1 (0.1)	0 (0)	6 (2.1)	5 (5.2)	2 (50)	< 0.001
Zero diastole ICA	0 (0)	0 (0)	0 (0)	1 (1)	0 (0)	0.076
Zero diastole CCA + ICA	0 (0)	0 (0)	0 (0)	1(1)	0 (0)	0.076
Diastolic reversal CCA	2 (0.2)	0 (0)	28 (9.9)	12 (12.5)	2 (50)	< 0.001
Diastolic reversal ICA	1 (0.1)	0 (0)	14 (5)	8 (8.3)	1 (25)	< 0.001
Diastolic reversal CCA + ICA	0 (0)	0 (0)	3 (1.1)	3 (3.1)	1 (25)	< 0.001
No dicrotic notch CCA	1 (0.1)	0 (0)	44 (15.6)	30 (31.3)	1 (25)	< 0.001
No dicrotic notch ICA	0 (0)	0 (0)	3 (1.1)	11(11.5)	0 (0)	< 0.001
No dichrotic notch CCA + ICA	0 (0)	0 (0)	1 (0.4)	7 (7.3)	0 (0)	< 0.001

% of patients within each group, *n* number of patients, *AR* aortic valve regurgitation, *CCA* common carotid artery, *ICA*: internal carotid artery, *ECA* external carotid artery, *OR* ods ratio, *CI* confidence interval

Regarding ICA, ‘bisferious pulse’ remained the most common flow alteration (approx. 12% of patients), followed by ‘diastolic reversal’ (ca. 11%). Although flow alterations in ICA were less frequent in comparison to CCA, an increased appearance was associated with severity of the valvular pathology as well. ‘Bisferious pulse’ and ‘diastolic reversal’ showed a highly significant association with AR in all evaluated carotid parts (singular and combined, *p* < 0.001). In singular and combined evaluation of CCA and ICA, association of AR and ‘no dicrotic notch’ was significant as well (*p* < 0.001). Interestingly, if ECA was considered, correlation significance decreased (*p* 0.011). The flow pattern ‘zero diastole’ was found to be significantly associated (*p* < 0.001) with AR only in CCA.

Overall, sensitivity was low (0.2–15.56%) due to the high number of patients without AR in this model. The positive prediction value (PPV) was found to be  > 90% for all evaluated flow characteristics in all examined vessels, despite ‘diastolic reversal’ in ICA (PPV = 50%). Moreover, specificity was detected to be  > 99% for all examined parameters, underlining a high probability of absence of cardiac valve pathology in patients without flow pattern alterations. Negative prediction value (NPV) was found to be approximately 65% (63–66%) for all flow patterns.

**Fig. 4:**
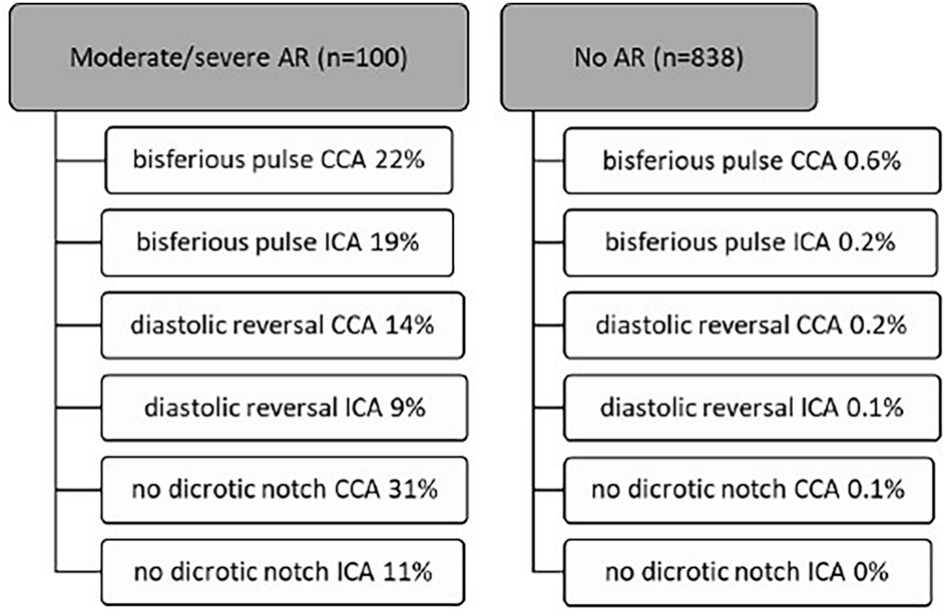
Distribution of Doppler flow curve characteristics in AR

To improve differentiation of patients with clinically significant cardiac valve pathology, patients without AR were compared to patients with at least moderate AR (including patients with moderate and severe AR, Fig. 4, Table 4, full data included in Additional file 1). Patients with minimal and mild AR were excluded from this analysis. In the group without vitium, no flow pattern changes were detected simultaneously in CCA and ICA. In patients with at least moderate AR, ‘no dicrotic notch’ was the most common flow deviation in the CCA (31% of patients) followed by ‘bisferious pulse’ (22%), ‘diastolic reversal’ (14%), whereas in ICA ‘bisferious pulse’ presented as the most common flow alteration (19%). Overall, flow pattern changes were more common in CCA than ICA, resulting in higher sensitivity of CCA compared to ICA. Nevertheless, the lowest sensitivity was detected for flow pattern changes in both vessels. Corresponding, positive predictive values were found to be close to 100% when assessing CCA and ICA, 90–100% in ICA and 81–97% in CCA. Specificity remained high, showing results of 100% specificity for combined evaluation of CCA and ICA, the lowest specificity was detected for singular analysis of CCA (99.4–99.88%).

**Table 4:** nvUS flow characteristics ‘no AR’ compared to at least ‘moderate AR’

Flow characteristic	No AR (*n*, %) *N* = 838	Moderate or severe AR (*n*, %) *N* = 100	*P* value	Sensitivity (%)	Specificity (%)	PPV (%)	NPV (%)
Bisferious pulse CCA	5 (0.6)	22 (22)	< 0.001	22	99.4	81.5	91.4
Bisferious pulse ICA	2 (0.2)	19 (19)	< 0.001	19	99.8	90.5	91.2
Bisferious pulse CCA + ICA	0 (0)	6 (6)	< 0.001	6	100	100	89.9
Zero diastole CCA	1 (0.1)	7 (7)	< 0.001	7	99.9	87.5	90
Zero diastole ICA	0 (0)	1 (1)	0.107	1	100	100	89.4
Zero diastole CCA + ICA	0 (0)	1(1)	0.107	1	100	100	89.4
Diastolic reversal CCA	2 (0.2)	14 (14)	< 0.001	14	99.8	87.5	90.7
Diastolic reversal ICA	1 (0.1)	9 (9)	< 0.001	9	99.9	90	90.2
Diastolic reversal CCA + ICA	0 (0)	4 (4)	< 0.001	4	100	100	89.7
No dicrotic notch CCA	1 (0.1)	31 (31)	< 0.001	31	99.9	98.7	93.4
No dicrotic notch ICA	0 (0)	11 (11)	< 0.001	11	100	100	90.4
No dicrotic notch CCA + ICA	0 (0)	7 (7)	< 0.001	7	100	100	90

Statistical analysis was performed using Chi-square test, Fisher’s exact test and fourfold table

*n* number of patient in each group, % percentage of patients in relation to number of patients in each group, *AR* aortic valve regurgitation, *CCA* common carotid artery, *ICA* internal carotid artery, *ECA* external carotid artery

### Doppler flow profile characteristics in patients with aortic valve stenosis

Patients without detection of aortic valve stenosis in echocardiogram showed ‘pulsus tardus et parvus’ in CCA in three cases (0.2%) (Table 5). Overall, 85% of patients with AS showed flow pattern changes in CCA and 75% in ICA. In combined evaluation of CCA and ICA, 53% of patients showed abnormal waveforms in nvUS. Moreover, frequency of nvUS alterations increased corresponding to the severity of the AS for all evaluated vessels (CCA, ICA, CCA + ICA, CCA + ICA + ECA). Interestingly, all patients with severe AS showed ‘pulsus tardus et parvus’ in nvUS in at least one evaluated vessel, displaying a significant correlation (*p* < 0.001) for AS and the appearance of ‘pulsus tardus et parvus’ in all examined parts of the carotid arteries.

**Table 5:** nvUS flow characteristics in patients with AS

Flow characteristic	No AS (*n*, %) *N* = 1245	Mild AS (*n*, %) *N* = 14	Moderate AS (*n*, %) *N* = 46	Severe AS (*n*, %) *N* = 15	*P* value
Pulsus tardus et parvus CCA	3 (0.2)	8 (57.1)	42 (91.3)	14 (93.3)	< 0.01
Pulsus tardus et parvus ICA	0 (0)	5 (35.7)	36 (78.3)	15 (100)	< 0.01
Pulsus tardus et parvus CCA + ICA	0 (0)	4 (28.6)	34 (73.9)	14 (93.3)	< 0.01
Pulsus tardus et parvus CCA + ICA + ECA	0 (0)	1 (7.4)	27 (58.7)	12 (80)	< 0.01

*n* number of patient in each group, % percentage of patients in relation to number of patients in each group, *AS* aortic valve stenosis, *CCA* common carotid artery, *ICA* internal carotid artery

Sensitivity was found to be highest in CCA (85%) and decreased slightly in ICA (75%). The lowest sensitivity (53%) was found for a combined analysis of CCA, ICA and ECA, with a positive predictive value of 100%. Sensitivity increased when only CCA and ICA were considered (69%), underlining reduced sensitivity for flow pattern deviations in ECA. A lack of flow pattern changes was detected with a specificity of ca. 100%, negative prediction values were found to be between 97 and 99%.

**Fig. 5:**
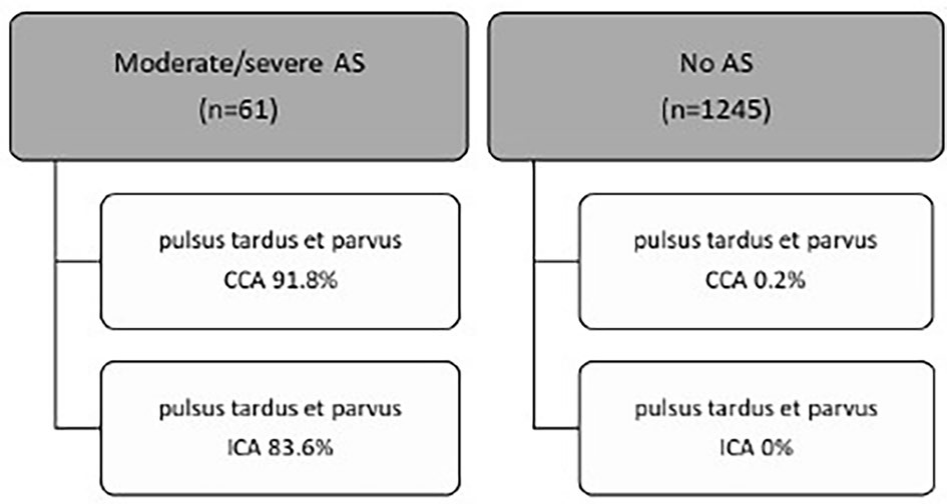
Distribution of Doppler flow curve characteristics in AS

When data for patients with at least moderate AS and severe AS was evaluated together in comparison to absence of AS in echocardiogram, ‘pulsus tardus et parvus’ was found to be apparent most often in CCA (92% of patients) (Table 6, Fig. 5). In ICA, 84% of patients showed a flow deviation, whereas nvUS delivered conspicuous results in both CCA and ICA in 79% of patients with AS. Statistical analysis revealed a highly significant correlation between AS and the appearance of ‘pulsus tardus et parvus’ in all evaluated parts of the carotid vessels (*p* < 0.001). Sensitivity increased to 92% in CCA and 84% in ICA, whereas specificity and negative predictive value remained mainly unchanged, underlining a good prediction probability.

**Table 6:** nvUS flow characteristics in patients with ‘no AS’ compared to at least ‘moderate AS’

Flow characteristic	No AS (*n*, %) *N* = 1245	Moderate or severe AS (*n*, %) *N* = 61	*P* value	Sensitivity (%)	Specificity (%)	PPV (%)	NPV (%)
Pulsus tardus et parvus CCA	3 (0.2)	56 (91.8)	< 0.001	91.8	99.8	94.9	99.6
Pulsus tardus et parvus ICA	0 (0)	51 (83.6)	< 0.001	83.6	100	100	99.2
Pulsus tardus et parvus CCA + ICA	0 (0)	48 (78.7)	< 0.001	78.8	100	100	98.9

Statistical analysis was performed using Chi-square test, Fisher’s exact test and fourfold table

*n* number of patients in each group, % percentage of patients in relation to number of patients, *AS* aortic valve stenosis, *CCA* common carotid artery, *ICA* internal carotid artery, *ECA* external carotid artery

### Statistical relationship between aortic valve regurgitation and nvUS flow profile changes

A logistic regression analysis was performed to evaluate causality between nvUS flow pattern changes and cardiac valve disease (Table 7). Patients without AR and minimal-to-mild AR were compared to a cohort of patients with moderate or severe AR.

**Table 7:** Results of regression analysis for aortic valve regurgitation

Flow characteristic	Odds ratio (OR) (confidence interval (CI))	*P* value
Bisferious pulse CCA	6.4 (3.4–11.9)	< 0.001
Zero diastole CCA	3.8 (1.1–13.8)	0.004
Diastolic reversal CCA	5.7 (2.7–12.2)	< 0.001
No dicrotic notch CCA	9.9 (5.6–17.4)	< 0.001
Bisferious pulse ICA	10.3 (5.4–19.7)	< 0.001
Diastolic reversal ICA	9.3 (3.8–22.9)	< 0.001
No dicrotic notch ICA	63.1 (17.0–234.3)	< 0.001
Bisferious pulse ECA	3.3 (1.3–8.3)	0.013
Zero diastole ECA	6.0 (2.8–12.8)	< 0.001
Diastolic reversal ECA	5.2 (2.6–10.6)	< 0.001
No dicrotic notch ECA	15.6 (7.6–32.1)	< 0.001
Bisferious pulse CCA + ICA	10.7 (3.4–33.9)	< 0.001
Diastolic reversal CCA + ICA	15.4 (3.2–74.6)	< 0.001
No dicrotic notch CCA + ICA	102.1 (12.4–839.4)	< 0.001

*CCA* common carotid artery, *ICA* internal carotid artery, *ECA* external carotid artery, *OR* ods ratio, *CI* confidence interval

All evaluated flow pattern characteristics were predictive for the existence of at least moderate AR. Highest predictive value was found for ‘no dicrotic notch’, especially when detected in CCA and ICA simultaneously, therefore, ‘no dicrotic notch’ seems to possess the most causal relationship with AR. The flow characteristic ‘diastolic reversal’ was associated with AR in all parts of the carotid arteries and showed a high predictive value in the ICA and in the CCA. Interestingly when CCA and ICA are assessed combined, odds ratio for ‘diastolic reversal’ was higher than for ‘bisferious pulse’. ‘Bisferious pulse’ delivered the highest predictive value when detected either in CCA and ICA or singularly in ICA. Overall, the flow characteristic ‘zero flow’ showed the least predictive value for AR. Analysis of ICA and combination of CCA and ICA was not possible due to insufficient case numbers displaying this flow pattern alteration in ICA. To conclude, the described characteristic flow patterns appear in increased frequency associated with the severity of AR, especially in CCA and ICA. Although flow deviations are more common in CCA, assessment of ICA increases specificity, which is underlined by the increased odds ratio in ICA and in the combined evaluation of both vessel parts.

### Statistical relationship between aortic valve stenosis and nvUS flow profile changes

Regression analysis for the flow pattern ‘pulsus tardus et parvus’ in nvUS and AS was performed, showing a significant relationship (*p* < 0.001) between the cardiac vitium and altered Doppler flow profile in carotid vessels. Patients with at least moderate AS were compared to patients without or mild AS (Table 8). Odds ratio for ‘pulsus tardus et parvus’ and AS was found to be high, especially if the flow profile was detected in CCA, ICA or both vessels, showing a high predictive value of this flow alteration for AS. Although assessment of ECA delivered a slightly lower predictive value compared to the other parts of the carotid vessels, a combined analysis of all vessel parts (CCA + ICA + ECA) yielded the highest predictive value.

**Table 8:** Results of regression analysis for aortic valve stenosis

Flow characteristic	Odds ratio (OR) (confidence interval (CI))	P value
Pulsus tardus et parvus CCA	1270.7 (427.0–3781.3)	< 0.001
Pulsus tardus et parvus ICA	1279.1 (421.8–3878.9)	< 0.001
Pulsus tardus et parvus ECA	812.1 (262.4–2513.6)	< 0.001
Pulsus tardus et parvus CCA + ICA	1158.5 (364.2–3684.8)	< 0.001
Pulsus tardus et parvus CCA + ICA + ECA	2230.1 (293.1–16966.4)	< 0.001

*CCA* common carotid artery, *ICA* internal carotid artery, *ECA* external carotid artery, *OR* ods ratio, *CI* confidence interval

### Inter-rater reliability

Twenty patients enrolled in the study were randomly chosen and the Doppler flow curve images were judged by a second rater with substantial (> 10 years) experience in neurovascular ultrasound. The second rater was blinded for the TEE and clinical data as well. Inter-rater reliability for the right and left common carotid artery was substantial (κ 0.671, *p* < 0.001 and κ 0.735, *p* < 0.001, respectively) as well as moderate for the right and left internal carotid artery (κ 0.467, *p* = 0.001 and κ 0.457, *p* < 0.001, respectively).

## Discussion

In the evaluated cohort of patients with TIA and ischemic stroke without occlusions or stenosis of the carotid vessels, doppler flow characteristics in the ICA and CCA were found to be highly predictive for aortic valve disease. This is in line with previous trials [7, 16] evaluating the effect of aortic valve pathologies on Doppler flow spectrums in nvUS. In contrast to prior studies, in this trial a larger number of patients were included, assessing the translatability of small cohort findings onto a larger scale. It has to be noted, that in the evaluated cohort, aortic valve stenosis was comparatively scarce (75 of 1320 patients, 5.7%), especially considering prior studies conducted in Europe, the USA and Taiwan, placing the population prevalence of AS at 12.4% with a prevalence of 3.4% of severe AS in people aged above 75 years [17]. It has to be considered that carotid artery disease is associated with aortic stenosis, due to similar pathomechanisms and probable causal relationship between both entities [18, 19]. One study showed prevalence of carotid plaque exceeding 90% in patients requiring aortic valve implantation, with carotid stenosis (> 50%) in 30% of patients [20]. Therefore, due to exclusion of patients with carotid vessel disease, the number of patients with aortic valve stenosis was possibly diminished in this study. The increased prevalence of AS in higher aged populations is in line with prior epidemiological findings [21].

In regard to baseline characteristics, patients assessed in this study displayed a typical profile of cardiovascular risk factors associated with ischemic stroke as a well as heart disease [22, 23]. Evaluation of baseline characteristics confirmed that in patients with aortic valve pathologies ischemic stroke is a common cardiovascular risk factor, underlining the interlinked relationship between both entities. Interestingly, in AR, age and severity of AR correlated, as described in literature [24], except for patients with severe AR. A possible explanation is an increased risk of stroke in this patient cohort at a younger age, possibly due to the aortic valve disease or to common risk factors for both diseases. Therefore, on average, patients with severe AR might suffer from ischemic stroke earlier in life compared to patients without or with mild/moderate AR. This would naturally influence the age of patients included into this study showing severe AR [25]. It must be noted that only four patients with severe aortic valve regurgitation were detected in this patient cohort, which could present a cofounding factor in the statistical analysis.

Flow profile changes in nvUS of carotid vessels were determined to be highly predictive of aortic valve disease and detected more often with increased severity of AR, due to corresponding increased reverse blood flow into the left ventricle. The flow patterns ‘no dicrotic notch’, ‘diastolic reversal’ and ‘bisferious pulse’ were observed more often than ‘zero diastole’. Different observations were made between CCA, ICA and ECA. Patients without AR showed flow profile changes only in singular cases and never both in CCA and ICA. These changes could possibly be caused by false negative TTE/TEE overlooking mild AR. Most common changes for AR were ‘bisferious pulse’ and ‘no dicrotic notch’ in CCA, in 50% of cases accompanied by ‘zero flow’/’diastolic reversal’. Although flow pattern changes in ICA were less frequent compared to the CCA, their appearance was more sensitive to a higher degree of severity of AR. Specificity overall was  > 99%. To conclude, the combined assessment of CCA and ICA increases sensitivity for detection of AR, with specificity remaining high. An assessment of ECA is less likely to contribute to a correct diagnosis, probably due to its inherent flow profile characteristics. For example, ‘bisferious pulse’ was found to be less predictive for detection of AR in ECA, whereas appearance of ‘no dicrotic notch’ in ECA was more predictive than in CCA. This alludes to the fact, that some flow profile changes are easier to detect in different parts of the carotid vessels than others, probably due to preexisting flow profile conditions [9–11].

In patients with AS, flow pattern alterations in nvUS were highly predictive for detection of aortic valve pathology as well. Using logistical regression analysis, the predictive value of nvUS is found to be increased with increased severity of AS. Higher percentage of flow pattern alterations in AS compared to AR might be caused by different pathomechanisms involved in the development of these changes. Reduced blood ejection due to AS is possibly translated more directly into carotids, whereas regurgitation phenomena are not reflected equally in carotid blood flow. Interestingly, sensitivity of flow profile analysis in nvUS increased for AS when CCA and ICA were evaluated without ECA. Possibly due to higher pulsatility in ECA, the flow pattern ‘pulsus tardus et parvus’ is less apparent. This underlines the importance of preexisting flow pattern characteristics in the assessed vessel structures. However, increased peripheral resistance can result in increased pulsatility in ICA and CCA as well. For example, carotid stenosis in distal parts of the vessel or microangiopathic cerebrovascular disease can increase ICA pulsatility, presenting a possible cofounder of flow pattern analysis in nvUS [26–28]. This needs to be considered when nvUS flow pattern evaluation is performed. High vigilance is especially needed when flow pattern changes are detected unilaterally: one Korean case report in 2019 detected ‘pulsus tardus et parvus’ associated with proximal CCA subocclusion, normalizing after stent implantation [29].

Differences in flow pattern changes for different parts of the carotid vessels were detected, as described extensively. This might be due to differences in distal transmission of effects of the cardiac vitium. As discussed, changes were found in a higher frequency in CCA than ICA, possibly due to proximity of CCA to the heart. Interestingly, in AR, appearance of flow pattern changes in ICA was associated with an increased severity of AR, supporting the hypothesis that a more severe aortic valve pathology results in increased distal transmission of blood flow changes. Moreover, transmission of the flow changes seems not only to be dependent on severity of AR, but is also different for various flow pattern changes. For example, ‘bisferious flow’ was the most frequent alteration in CCA and ICA, whereas ‘no dicrotic notch’ was more common in CCA than in ICA, suggesting the ‘no dicrotic notch’ pattern to be less transmittable than ‘bisferious pulse’. To conclude, for analysis and screening for aortic valve pathologies in patients with ischemic stroke/TIA, ICA, preferably in combination with CCA, should be assessed, offering best sensitivity and predictive value.

Neurovascular ultrasound is a cheap, safe, and easily accessible diagnostic tool that can be performed in an outpatient setting. It is usually well tolerated by patients [30] and has no negative side effects. Our data show that changes in carotid flow profiles in nvUS are highly predictive for aortic valve disease and can be supportive in early identification of at-risk patients. The consideration of the described flow characteristics can be useful in streamlining of diagnostics and therapeutic measures in ischemic stroke patients, identifying high-risk patients for fast-tracking of additional diagnostics. Additionally, awareness of possible aortic valve pathologies influencing nvUS results allows improved interpretation of detected flow alterations. Since aortic valve pathology can influence results of cerebral vessel diagnostics, awareness of flow pattern changes can support interpretation of neurovascular ultrasound results, especially in neurocritical ill patients. For example, flow pattern changes in epiaortic arteries can be useful for contextualizing transcranial color-coded sonography (TCCD) results (e.g., low mean flow velocity and low diastolic flow velocity associated with severe aortic regurgitation compared to low cerebral perfusion due to increased intracranial pressure [31]), providing an at-hand, bedside tool within the same diagnostic procedure. Nevertheless, cardiac ultrasound is indispensable and cannot be substituted by flow pattern analysis in nvUS since TTE/TEE provides valuable and necessary information that cannot be gained through nvUS of the carotid vessels. Naturally, cardiac ultrasound should therefore be performed in all patients with ischemic stroke/TIA, whereas flow pattern analysis in nvUS provides an additional diagnostic measure improving clinical workup.

### Limitations and strengths of the study

The main limitation is the retrospective nature of the described study. Firstly, neurovascular ultrasound images were obtained during routine diagnostics; therefore, their quality depends on the examinator, and no on-patient re-analysis was possible. However, bias of examinator performing nvUS is not a relevant concern since images were not obtained with flow pattern analysis in mind. Due to the retrospective study design, patient information and diagnosis were not verifiable, and missing data were unable to be completed subsequently, therefore patients with incomplete data sets were not included into the study. Additionally, interpretation of the study is limited through exclusion of patients with carotid vessel pathologies (e.g., stenosis or occlusion). Due to similar risk factor profiles, patients with severe aortic valve pathology might be excluded from the cohort which could alter the results. Despite the limitations of the observational retrospective study design, it allowed inclusion of a large cohort of patients with ischemic stroke / TIA in this study, translating findings of small prior studies onto a larger scale and providing reliable statistical analysis. To our knowledge, this is the first study evaluating flow pattern changes in nvUS associated with aortic valve pathology in a large cohort. Moreover, epiaortic artery ultrasound was performed by trained clinicians during routine diagnostics, supporting sufficient quality of obtained images and reducing bias regarding study objectives. Image raters were blinded for TTE/TEE results and did not physically see the patient, therefore no bias resulting from clinical signs of aortic valve pathology must be accounted for.

## Conclusion

Aortic valve disease and ischemic stroke/TIA present relevant comorbidities due to a shared risk factor profile. Neurovascular ultrasound is a highly accessible, safe diagnostic method that is part of the routine clinical work up in ischemic stroke/TIA and can be easily performed in an outpatient setting. In this study, evaluation of flow profile changes in nvUS of CCA and ICA in patients without carotid artery pathology was highly predictive for detection of AS and AR. Assessment should include nvUS of ICA and CCA, whereas ECA is not as suitable for flow profile evaluation. Clinicians performing nvUS should be familiar with the described flow profiles and their implications, prioritizing at-risk patients for aortic valve pathology for echocardiography as well as improving interpretation of nvUS results. Although, epiaortic artery ultrasound does not substitute for TTE/TEE diagnostics in patients with ischemic stroke / TIA, it provides a supportive additional measure increasing accuracy of clinical examination.

## Supplementary Information

**Additional file 1: Table S1. **Complete nvUS flow characteristics in patients with AR. **Table S2.** Complete nvUS flow characteristics ‘no AR’ compared to at least ‘moderate AR’.

## Data Availability

All data generated or analyzed during this study are included in this published article.

## Abbreviations

AR: Aortic valve regurgitation
AS: Aortic valve stenosis
CCA: Common carotid artery
CI: Confidence interval
ECA: External carotid artery
ICA: Internal carotid artery
NPV: Negative predictive value
nvUS: Neurovascular ultrasound
OR: Odds ratio
PAD: Peripheral artery disease
PPV: Positive predictive value
TEE: Transesophageal echocardiogram
TIA: Transient ischemic attack
TTE: Transthoracic echocardiogram
