# Supplementary material for: Doppler flow morphology characteristics of epiaortic arteries in aortic valve pathologies: a retrospective study on a cohort of patients with ischemic stroke
Source: Ultrasound J. 2023 Jun 7;15:29. doi: 10.1186/s13089-023-00327-4 (PMC10247641; doi:10.1186/s13089-023-00327-4)
Supplement: Supplementary file 1 — Additional file 1: Table S1. Complete nvUS flow characteristics in patients with AR. Table S2. Complete nvUS flow characteristics ‘no AR’ compared to at least ‘moderate AR’. [file 13089_2023_327_MOESM1_ESM.pdf]

Supplementary data:

Flow characteristic	No AR (n, %) N = 838	Minimal AR (n, %) N = 100	Mild AR (n, %) N = 282	Moderate AR (n, %) N = 96	Severe AR (n, %) N = 4	P value	Sensitivity (%)	Specificity (%)	PPV (%)	NPV (%)
Bisferious pulse CCA	5 (0,6)	0 (0)	40 (14,2)	21 (21,9)	1 (25)	< 0,001	12,86	99,4	92,54	66,48
Bisferious pulse ICA	2 (0,2)	1 (1)	27 (9,6)	18 (18,8)	1 (25)	< 0,001	9,75	99,7	95,91	65,77
Bisferious pulse CCA + ICA	0 (0)	0 (0)	7 (2,5)	5 (5,2)	1 (25)	< 0,001	2,69	100	100	64,12
Bisferious pulse CCA + ICA + ECA	0 (0)	0 (0)	2 (0,7)	2 (2,1)	1 (25)	< 0,001	10,37	100	100	63,73
Zero diastole CCA	1 (0,1)	0 (0)	6 (2,1)	5 (5,2)	2 (50)	< 0,001	2,69	99,88	92,86	64,09
Zero diastole ICA	0 (0)	0 (0)	0 (0)	1 (1)	0 (0)	0,076	0,2	100	100	63,53
Zero diastole CCA + ICA	0 (0)	0 (0)	0 (0)	1(1)	0 (0)	0,076	0,2	100	100	63,53
Diastolic reversal CCA	2 (0,2)	0 (0)	28 (9,9)	12 (12.5)	2 (50)	< 0,001	8,71	99,76	95,45	65,52
Diastolic reversal ICA	1 (0,1)	0 (0)	14 (5)	8 (8,3)	1 (25)	< 0,001	0,2	99,88	50	63,51
Diastolic reversal CCA + ICA	0 (0)	0 (0)	3 (1,1)	3 (3,1)	1 (25)	< 0,001	1,45	100	100	63,82
Diastolic reversal CCA + ICA + ECA	0 (0)	0 (0)	0 (0)	2 (2,1)	1 (25)	< 0,001	0,6	100	100	63,63
No dicrotic notch CCA	1 (0,1)	0 (0)	44 (15,6)	30 (31,3)	1 (25)	< 0,001	15,56	99,88	98,68	67,28
No dicrotic notch ICA	0 (0)	0 (0)	3 (1,1)	11(11,5)	0 (0)	< 0,001	2,9	100	100	64,17
No dictrotic notch CCA + ICA	0 (0)	0 (0)	1 (0,4)	7 (7,3)	0 (0)	< 0,001	1,66	100	100	63,87
No dicrotic notch CCA + ICA + ECA	0 (0)	0 (0)	0 (0)	2 (2.1)	0 (0)	0,011	0,4	100	100	63.63
Statistical analysis was performed using chi-square test, fishers exact test and fourfold table										
N: absolute number of patients with each aortic valve pathology										
n: number of patient in each group, % percentage of patients in relation to number of patients in each group										

Suppl. Table 1: complete nvUS flow characteristics in patients with AR

Flow characteristic	No AR (n, %) N = 838	Moderate or severe AR (n, %) N = 100	P value	Sensitivity (%)	Specificity (%)	PPV (%)	NPV (%)
Bisferious pulse CCA	5 (0,6)	22 (22)	< 0,001	22	99,4	81,48	91,44
Bisferious pulse ICA	2 (0,2)	19 (19)	< 0,001	19	99,76	90,48	91,17
Bisferious pulse CCA + ICA	0 (0)	6 (6)	< 0,001	6	100	100	89,91
Zero diastole CCA	1 (0,1)	7 (7)	< 0,001	7	99,88	87,5	90
Zero diastole ICA	0 (0)	1 (1)	0,107	1	100	100	89,43

Zero diastole CCA + ICA	0 (0)	1(1)	0,107	1	100	100	89,43
Diastolic reversal CCA	2 (0,2)	14 (14)	< 0,001	14	99,76	87,5	90,67
Diastolic reversal ICA	1 (0,1)	9 (9)	< 0,001	9	99,88	90	90,19
Diastolic reversal CCA + ICA	0 (0)	4 (4)	< 0,001	4	100	100	89,72
No dicrotic notch CCA	1 (0,1)	31 (31)	< 0,001	31	99,88	98,68	93,38
No dicrotic notch ICA	0 (0)	11 (11)	< 0,001	11	100	100	90,4
No dictrotic notch CCA + ICA	0 (0)	7 (7)	< 0,001	7	100	100	90
Statistical analysis was performed using chi-square test, fishers exact test and fourfold table							
N: absolute number of patients with each aortic valve pathology							
n: number of patient in each group, % percentage of patients in relation to number of patients in each group							

Suppl. Table 2: complete nvUS flow characteristics 'no AR' compared to at least 'moderate AR'
